# Contraceptive adoption in the extended postpartum period is low in Northwest Ethiopia

**DOI:** 10.1186/s12884-015-0598-9

**Published:** 2015-08-01

**Authors:** Zelalem Birhanu Mengesha, Abebaw Gebeyehu Worku, Senafikish Amsalu Feleke

**Affiliations:** Department of Reproductive Health, Institute of Public, University of Gondar, Gondar, Ethiopia

## Abstract

**Background:**

The extended postpartum period is a one year period after delivery which is critical for women to prevent unintended pregnancy and to reduce the risk of maternal and child mortality by ensuring safe birth intervals. Studies indicate that birth intervals of three to five years reduce maternal mortality and provide health benefits to newborn babies, infants, and children. As a result, assessing postpartum contraceptive use and its determinants are an increasingly important component of global health. The objectives of the study were to determine postpartum contraceptive use and identify the variables which affect postpartum contraceptive use among women of Dabat district.

**Methods:**

All women aged 15 to 49 years who delivered a child between January 1, 2012 and December 31, 2012 in the Debat district were interviewed by house-to- house survey.

**Results:**

A total of 10.3 % of the mothers reported adopting contraception in the extended postpartum period. Women who delivered with the assistance of a skilled attendant [AOR = 1.88, 95 % CI (1.01-3.51)] and attended postnatal care services [AOR = 2.19, 95 % CI (1.06-4.52)] were more likely to use contraceptives. Secondary and above level of the husband’s education was also a variable that significantly affected postpartum contraceptive use [AOR = 2.98, 95 % CI (1.49-5.97)].

**Conclusions:**

Contraceptive use in the extended postpartum period was found to be low placing women at risk for a pregnancy in the extended postpartum period. Advice about contraceptives during postnatal clinic visits was limited. Improving utilization of institutional delivery by a skilled attendant and enhancing postnatal care services are important to increase contraceptive use in the extended postpartum period.

## Background

The benefit of postpartum family planning (PPFP) for maternal and child survival has long been recognized and the concept of implementing special family planning programs for postpartum women has been recognized as the standard of care since 1966 [1]. Studies show that, the risk of maternal and child mortality and morbidity are very high when pregnancy occurs in short intervals after childbirth [2–7].

Globally, more than 90 percent of women during the first year of postpartum period want to either delay or avoid future pregnancies [8]. However, in most cases, sexual activity in the postpartum is resumed before the first menstruation following delivery without the use of any contraceptive method [9–11].

In sub-Saharan Africa, the proportion of postpartum women who are exposed to the risk of pregnancy by having sex while using no contraceptive method within 2 years after childbirth is nearly one third [9, 12]. Most postpartum women expressed a desire to prevent pregnancy during first two years after delivery but had not obtained contraceptive protection [13]. According to Demographic Health Surveys (DHS) in 27 countries, 92 to 97 percent of those women who did not want another child within two years after delivery, yet 35 % of them had their children spaced less than two years apart [8]. In addition, between 20 and 40 percent of women did not initiate contraception before they became at risk for another pregnancy and the percentage of women who did not adopt a method and became pregnant is highest in Kenya (31 %) and lowest in Indonesia (11 %) [14].

PPFP usage varies in sub-Saharan countries, which is 40 % in Zambia, 25 % in Kenya, 20 % in Tanzania, 15 % in Nigeria and less than 10 % in Ethiopia. In a recent study, women of postpartum period were less likely to use family planning by the end of the extended postpartum period when compared with married women in the general population. Those using contraception made up only a small proportion of those needing it [15].

The use of maternal health services like antenatal and postnatal care services, levels of education and exposure to family planning messages are some of the predictors for the use of modern contraception by women in the postpartum period [1]. There are also several reasons for the non use of PPFP, like unpredicted sex, relying on breast feeding, and lack of access [8, 16].

In Ethiopia, breast feeding is universal but only half (52 %) of children under six months are being exclusively/optimally breastfed [17]. Hence, the use of lactational amenorrhea cannot be effectively and reliably used as a method of contraception. Therefore, this research sought to investigate the patterns and factors associated with postpartum contraceptive use among women who gave birth one year preceding the survey in Dabat district.

## Methods

### Study design and study area

A community based cross-sectional study was conducted in January 2013 in Dabat district. Dabat district is one of the 21 districts in North Gondar Administrative Zone of Amhara Region, Ethiopia. According to the 2007 census report, the district had an estimated total population of 145,458 living in thirty kebeles (lowest administrative units) and the local communities largely depend on subsistent agricultural economy. Amhara is the largest ethnic group in Dabat (99.4 %). About 97.7 % of the district population are Orthodox Christians and 89 % of the population lived in rural areas [18]. Each kebele has a health post to provide family planning services for about 1000 households. We selected this particular area because it is being used as a surveillance site with ten selected kebeles for the University of Gondar in which a continuous monitoring and updating of subsequent vital events like births, deaths and migration are being recorded. The study site consists of seven rural and three urban kebeles [19].

### Study population and sampling

The study population was all women aged 15 to 49 years who delivered a baby between January 1, 2012 and December 31, 2012 in Debat district. Sample size was determined using the single population proportion formula considering the following assumptions: 95 % confidence level, proportion of 29 % [17], marginal error of 4 %, and design effect of 1.5. Sample size was also increased by 10 % for non-response. This makes the number of women to be interviewed 816. However, during data collection in the field, we considered all women who gave birth in the period from January 1, 2012 to December 31, 2012 and we got 899 samples. Finally we interviewed all the 899 women for this study.

### Data collection

A structured questionnaire first prepared in English and translated to the local language (Amharic) was used to collect data. The questionnaire was pretested among 45 women living in the kebeles that are not part of the surveillance centre and revisions were made before the actual data collection. During the interviews, maternal health service utilization, fertility, and contraceptive use were recorded. All the seventeen data collectors and the three onsite supervisors of the research center were employed. Data quality was maintained via intensive training of the data collectors and supervisors, pretesting of data collection tools and close supervision by resident supervisors and principal investigators.

### Data processing and analysis

Experienced clerks entered the data using EPI Info 3.5.3 statistical software. After checking for consistency and completeness, data were exported to SPSS [20] for further analysis. Multiple logistic regression analysis was used to identify variables independently associated with postpartum contraceptive use. All variables with p value of less than 0.02 in the bivariate analysis were considered for multivariate analysis. The strength of the association was interpreted using the adjusted odds ratio with 95 % CI. The criterion for statistical significance was set at p value of 0.05. Tables and texts were used to present the findings of the study.

### Ethical consideration

Before the commencement of the study, the Institutional Review Board of the College of Medicine and Health Sciences of the University of Gondar, reviewed and approved the study protocol. During data collection, all study subjects were asked for consent and all participants signed consent forms. To ensure confidentiality, names were not used in depicting the results of the study.

## Results

### Socio-demographic characteristics

We interviewed 899 women for this study. The mean age of the study participants was 28.3 ± 6.4. Nearly half (47.5 %) of the respondents were between the age of twenty-five and thirty-four years with equal proportions younger and older. Approximately three-quarters (74.2 %) of the respondents resided in rural kebeles of the study area. Almost all of the women (99.7 %) were from the Amhara ethnic group. Ethiopian Orthodox Christians accounted for 96.6 %. Most (93 %) were married while the remaining women were unmarried (2.9 %), divorced (2.9 %), separated (1.0 %), or widowed (0.2 %) (Table 1).

**Table 1:** Socio-demographic characteristics of the study participants (N = 899), January 2013

Variable		Frequency	Percentage (%)
Age (years)	15-24	269	29.9
	25-34	427	47.5
	>_35	203	22.6
Residence	Urban	232	25.8
	Rural	667	74.2
Ethnicity	Amhara	896	99.7
	Tigrie	3	0.3
Religion	Orthodox	868	96.6
	Muslim	28	3.1
	Catholic	3	0.3
Marital status	Married	836	93.0
	Unmarried	26	2.9
	Widowed	2	.2
	Divorced	26	2.9
	Separated	9	1.0
Educational status	Cannot read and write	579	64.4
	Read and write	41	4.6
	Primary(1–8)	145	16.1
	Secondary(9–12)	118	13.1
	Higher education	16	1.8
Husband education	Cannot read and write	355	39.5
	Read and write	228	25.4
	Primary(1–8)	174	19.4
	Secondary(9–12)	103	11.5
	Higher education	32	3.6
Maternal occupation	Farmer	634	70.5
	Merchant	20	2.2
	Govt employee	34	3.8
	Others	211	23.5
Husband occupation	Farmer	716	79.6
	Merchant	33	3.7
	Govt employee	88	9.8
	Others	48	5.3

The majority (64.4 %) of the mothers could not read or write and 145 (16.1 %) had attended primary school, 118 (13.1 %) secondary school, and 16 (1.8 %) had attended tertiary education. With their husbands, 39.5 % could not read or write, 25.4 % could read and write, 19.4 % had attended primary education, and 11.5 % had secondary education and 3.6 % tertiary education. The most frequently reported occupation by both the mother and husband was farmer at 70.5 % and 79.6 %, respectively (Table 1).

### Use of maternal health services during the last pregnancy

A total of 468 or 52.1 % had attended antenatal clinic (ANC) services and 329 (36.6 %) had attended between two and four visits. Sixty women (6.7 %) had attended five or more visits. ANC visits were most frequently conducted at the health center (41.4 %) and health posts (9.2 %). A nurse-midwife was the most frequent type of ANC attendant followed by health extension workers. The home was the most frequent place of delivery at 81.1 %. The health center followed at 15.8 %. Twenty-six or 2.9 % delivered in a hospital and 81.1 % of the babies were delivered by non-skilled attendants. A total of fifty-one mothers or 5.7 % sought postnatal care (PNC). The postnatal visits were most frequently held at the health center. A nurse-midwife was the attendant for thirty-seven of the fifty-one mothers. Twenty-six percent were advised about contraception during their PNC visits (Table 2).

**Table 2:** Maternal health service utilization during the last pregnancy, Jan 2013

Variable		Frequency	Percentage (%)
ANC	Yes	468	52.1
	No	431	47.9
Frequency of ANC	No	433	48.2
	One	77	8.6
	two-four	329	36.6
	Five and above	60	6.7
Place of ANC	Health Post	83	9.2
	Health Center	372	41.4
	Hospital	4	.4
	Others	9	1.0
Type of attendant	Health Extension Workers	81	9.0
	Nurse/Midwife	372	41.4
	Health Officer	7	.8
	Doctor	8	.9
Place of delivery	Home	729	81.1
	Health Post	2	.2
	Health Center	142	15.8
	Hospital	26	2.9
Assistance during delivery	Non Skilled	729	81.1
	Skilled	170	18.9
PNC	Yes	51	5.7
	No	848	94.3
Place of PNC	Health Center	38	4.2
	Hospital	8	.9
	Others	5	.6
Type of attendant	TBA	1	.1
	HEW	2	.2
	Nurse/Midwife/Health Officer	41	4.5
	Doctor	7	.8
Advise about contraceptives during PNC	Yes	242	26.9
	No	582	64.7

*ANC* Antenatal Care

*PNC* Postnatal Care

### Contraception history

A total of 348 (38.7 %) had used contraceptives prior to the recent birth. The most frequently used contraceptive method was the injection (30.6 %) and birth control pill (9.9 %). Ten percent were currently using a contraceptive and among these women, the injection was the most popular method. The most frequently cited reason for using a contraceptive was spacing children. The government health center was the primary source for contraceptives and partners were aware of contraceptive use in most cases. Most women (83.1 %) intended to use contraception in the future. The decision about contraceptive use was reported as a joint decision made by both partners (Table 3).

**Table 3:** Contraceptive history and current behavior of the study participants, Jan 2013

Variable		Frequency	Percentage (%)
Previous Use of Contraceptives	Yes	348	38.7
	No	551	61.3
Type of contraceptive	Injection	275	30.6
	Implanolol	13	1.4
	IUCD	1	0.1
	Condom	2	0.2
	Pills	89	9.9
	Natural method	2	0.2
Current Use of Contraceptives	Yes	93	10.3
	No	806	89.7
Type of contraceptive	Injection	74	8.2
	Pills	3	0.3
	IUCD	16	1.8
	Condom	1	0.1
	Implanolol	2	0.2
Reason for use	Limiting	28	3.1
	Spacing	64	7.1
Source of contraceptives	Hospital	1	.1
	Health center	64	7.1
	Health post	12	1.3
	Private clinic	16	1.7
Using the preferred method	Yes	88	9.8
	No	4	.4
Partner awareness	Yes	83	9.2
	No	9	1.0
Intention to use in the future	Yes	747	83.1
	No	152	16.9
Reason to use	Limiting	445	49.5
	Spacing	302	33.6
Decision maker about contraceptive	Me myself	257	28.6
	Partner	10	1.1
	Both	632	70.3

### Determinants of contraceptive use

As clearly shown on the multivariate logistic regression Table 4, urban residence, secondary and above level of husband’s education, delivery by a skilled attendance and PNC follow up were the factors significantly and independently associated with contraceptive use among women in the extended post partum period.

**Table 4:** Predictors of postpartum contraceptive use at Debat DSS site, Northwest Ethiopia, 2013

Determinants		Contraceptive use		Crude OR (95 % CI)	Adjusted OR (95 % CI)
		Yes	No		
Residence	Urban	74	158	15.97 (9.37-27.23)	5.83 (2.93-11.63)
	Rural	19	648	Ref	Ref
Age in years	15-24	34	235	1.95 (1.02-3.75)	
	25-34	45	382	1.59 (.85-2.97)	
	>_35	14	189	Ref	
Number of living children	0-2	58	250	Ref	
	3-4	24	270	6.03 (3.09-11.75)	
	5+	11	286	2.31 (1.11-4.81)	
ANC follow up	0	12	421	Ref	
	1	6	71	2.97 (1.08-8.15)	
	2-4	54	275	6.89 (3.62-13.11)	
	≥5	21	39	18.89 (8.65-41.27)	
ANC attendant	HEW	2	79	.88 (.19-4.03)	
	Nurse/HO/Midwife	79	308	8.96 (4.79-16.73)	
	No attendant	12	419	Ref	
Husband’s Education	No formal education	25	558	Ref	Ref
	Primary education	13	161	1.80 (.90-3.60)	1.04 (.49-2.21)
	Secondary and above	54	81	14.88 (8.77-25.24)	2.98 (1.49-5.97)
Maternal Education	No formal education	26	594	Ref	
	Primary education	15	130	2.64 (1.36-5.12)	
	Secondary and above	52	82	14.49 (8.58-24.48)	
Delivery Assistance	Non skilled	36	693	Ref	Ref
	Skilled	57	113	9.71 (6.12-15.42)	1.88 (1.008-3.51)
PNC	Yes	19	32	6.21 (3.36-11.49)	2.19 (1.06-4.52)
	No	74	774	Ref	Ref

*ANC* Antenatal Care

*PNC* Postnatal Care

Postpartum contraceptive use was higher amongst females from urban areas [AOR = 5.83, 95 % CI (2.93, 11.63)]. Secondary and above level of husband’s education was also observed to have a significant influence on utilization of family planning methods [AOR = 2.98, 95 % CI (1.49, 5.97)]. Women who had PNC visit were 2.19 times [AOR = 2.19, 95 % CI (1.06, 4.52)] more likely to use contraceptive as compared to those who didn’t have follow up. Delivery by a skilled attendant was found to be an important determinant of contraceptive use in the extended post partum period in which women who delivered with the assistance of a skilled attendant were 1.88 times [AOR = 1.88, 95 % CI (1.01, 3.51)] more likely to use contraceptive as compared to those who didn’t receive skilled care during delivery (Table 4).

## Discussion

Postpartum family planning (PPFP) focuses on the prevention of unintended and closely spaced pregnancies through the first 12 months following childbirth [20]. Evidences show that family planning can prevent more than 30 % of maternal deaths and 10 % of child mortality if couples space their pregnancies more than 2 years apart [6]. In addition, data from 27 developing countries show that 95 % of postpartum women want to avoid a pregnancy for at least 2 years and 65 % of women who are 0–12 months post partum want to avoid a pregnancy in the next 12 months but are not using contraception [8]. Therefore studying the contraceptive practice of women in the extended post partum period has a paramount importance to improve contraceptive use in Ethiopia.

This study revealed that, the postpartum contraceptive method use was 10.3 %; this finding was much lower than the result of 2011 EDHS which reported 29.0 and 33 % for Ethiopian and Amhara region married women respectively [17]. This is because postpartum women may not realize they are at risk of pregnancy even if they are breastfeeding [21]. Among those who adopted postpartum contraception in our study, 8.2 % used Injectables while 1.8 % used IUD. Injectables and implants were the most common methods reported in the study during the 2011 EDHS [17].

The findings show that the use of a modern method of contraception during the postpartum period is significantly associated with use of maternal health services (Facility delivery and PNC) when maternal health care is disaggregated into ANC, institutional delivery and PNC services. This relationship is consistent with findings reported by other studies in India and Mexico [22, 23]. This can be attributed to the family planning advice received by the females at a hospital/health centre during facility delivery and post natal care service taking. The findings suggest that facility delivery and postnatal services remain important windows of opportunity to provide access to family planning messages and to offer women various contraceptive methods. Almost all of the women obtained maternal health services from the public sector. Therefore this finding underlines the needs for service integration within the public sector in order to take advantage of the delivery and postnatal period to increase the uptake of modern family planning during the critical postpartum period.

The utilization of antenatal care service was not found to be a significant predictor of the use of modern contraception in the postpartum period. This finding contrasts with those of some similar research in Mexico, which reported that women who had antenatal care service were more likely to use a contraceptive method postpartum than those who delivered at home [23].

In addition to the maternal health services utilization, other significant predictors of the use of contraception in the postpartum period include secondary and above levels of husband education, and urban residence. The findings show that the odds of using a modern method of contraception are relatively high among women from urban areas compared with the rural women. This is not surprising, as urban women tend to have better access to health facilities and other promotional activities that are usually urban based [24].

Present study showed significant association between husband’s education and use of contraceptives among women in the extended post partum period. Higher education of husbands promotes the use of contraceptives. Husband’s education was found significant in deciding use of contraceptive even with multivariate analysis. Similar findings were reported from studies done in Ethiopia and India [25, 26]. This finding highlights the importance of focusing on involving men in family planning efforts because husbands do seem to play a role in deciding family planning methods for their wives.

## Conclusions

The findings show that adoption of contraception in the extended post partum period is low in Northwest Ethiopia. The use of a modern method of contraception during the postpartum period was significantly associated with use of maternal health services (Facility delivery and PNC), secondary and above level of husband’s education and urban residence. Integration of family planning services with maternal health services is highly recommended to increase contraceptive use in the post partum period in Ethiopia.
